# Factors Associated with Cancer- and Non-Cancer-Related Deaths among Taiwanese Patients with Diabetes after 17 Years of Follow-Up

**DOI:** 10.1371/journal.pone.0147916

**Published:** 2016-12-01

**Authors:** Chin-Hsiao Tseng

**Affiliations:** 1 Department of Internal Medicine, National Taiwan University College of Medicine, Taipei, Taiwan; 2 Division of Endocrinology and Metabolism, Department of Internal Medicine, National Taiwan University Hospital, Taipei, Taiwan; 3 Division of Environmental Health and Occupational Medicine of the National Health Research Institutes, Taipei, Taiwan; Johns Hopkins University Bloomberg School of Public Health, UNITED STATES

## Abstract

**Objective:**

A previous 12-year follow-up of a large diabetes cohort in Taiwan suggested a survival advantage in the patients with obesity. The present study further investigated additional determinants for cancer and non-cancer death in the cohort after a follow-up of 17 years.

**Methods:**

A cohort of 92546 diabetes patients recruited since 1995 was followed for vital status by matching the National Death Certificate Database until 2011. Cox regression estimated the hazard ratios for the following variables: age at baseline, sex, diabetes type, screen-detected diabetes (diabetes diagnosed accidentally through epidemiological screening programs or during visits to medical settings without a history of diabetes), diabetes duration, body mass index, insulin use, hypertension, smoking, and living region. Fasting glucose and history of dyslipidemia were available for additional adjustment in a subcohort of the patients (n = 14559).

**Results:**

A total of 40229 diabetes patients (43.5% of the cohort) died during follow-up and 10.9% died under the age of 60. Insulin use and smoking significantly predicted cancer and non-cancer death. The adjusted hazard ratio (95% confidence interval) associated with insulin use was 1.161 (1.052–1.281) for cancer death and 1.469 (1.413–1.526) for non-cancer death. Screen-detected diabetes and body mass index were consistently associated with a lower risk, but diabetes duration a higher risk, for non-cancer death, with adjusted hazard ratio of 0.683 (0.666–0.702), 0.955 (0.951–0.958) and 1.018 (1.017–1.020), respectively. Diabetes type had a null association disregarding the causes of death and living in rural areas was significantly associated with a higher mortality from non-cancer death. Hypertension, fasting glucose and dyslipidemia showed differential impacts on cancer and non-cancer death, and were significantly predictive for non-cancer death.

**Conclusions:**

Screen-detected diabetes and a higher body mass index provide a survival advantage, especially for non-cancer death. However, insulin use is associated with a significantly higher risk of either cancer or non-cancer death.

## Introduction

Diabetes is a common non-communicable disease that affects hundreds of millions of people worldwide [1]. In the year 2015, the International Diabetes Federation estimated a total global number of 415 million people suffering from diabetes and 37% among them live in the Western Pacific region [1]. Nearly half of the people with diabetes do not know that they are having diabetes [1]. What is worse is that 5 million people died of diabetes in 2015, and nearly a half of them (46.6%) were under the age of 60 [1]. The incidence and prevalence of diabetes is on the rise [2–4] and the global prevalence of diabetes for all ages was estimated to rise from 2.8% in 2000 to 4.4% in 2030 [5]. The International Diabetes Federation estimated that there will be 642 million adults (aged 20–79 years) living with diabetes in 2040 [1].

In Taiwan, a series of epidemiological screening programs have been conducted during the past five decades [4]. In Taipei city, the prevalence of diabetes for residents aged 40 years or older has been increasing from 5.1% in 1970, to 7.1% in 1979 and to 8.2% in 1986 [4]. During 1993–1996 and 2005–2008, respectively, two cycles of the Nutrition and Health Survey in Taiwan (NAHSIT) based on similar sampling strategy were conducted among residents aged 19 years or older. The crude nation-wide prevalence of diabetes for residents aged 19 years or older increased from 5.33% (age-standardized rates: 6.21%) in the first NAHSIT survey to 9.05% (age-standardized rates: 7.80%) in the second NAHSIT survey [6]. The increased prevalence of diabetes was especially striking for the older population aged 65 years or older, from 17.13% to 25.73%, for the first and second survey, respectively [6]. Another national survey, the Taiwanese Survey on Hypertension, Hyperglycemia and Hyperlipidemia, was conducted in 2002, and from a random sample of 4683 residents aged 25–74 years, the crude prevalence of diabetes in men (9.1%) was significantly higher than that in women (5.6%) [7].

Patients with diabetes suffer from a high prevalence of comorbidities such as obesity, hypertension and dyslipidemia [7–10]. All of these contribute to the higher risk of developing cardiovascular disease, which remains as the most important cause of death in the patients [11]. Recent studies also strongly suggested a close link between diabetes and cancer risk in terms of incidence or mortality [11–17]. Additionally, a potential role of insulin was noted in the development of hypertension, atherosclerosis and cancer in patients with diabetes [10,13,18–20]. In a recent 12-year follow-up of a cohort of Taiwanese patients with type 2 diabetes, in contrary to the general concept of an increased risk of mortality related to obesity, a survival advantage was observed in the obese diabetic patients [21]. When cancer and non-cancer causes of death were analyzed separately, such a survival advantage with obesity was mainly observed in non-cancer mortality [21]. Another controversial but interesting and important issue is that whether screen-detected diabetes may provide survival advantage for the patients [22–25]. Therefore, identification of determinants for the mortality in patients with diabetes is important for an effective intervention to curb the increasing trends of diabetes-related mortality.

By continuously following the large nationally representative cohort of patients with diabetes recruited since 1995 in Taiwan [21], the present study aimed at evaluating predictors for mortality in the patients after a follow-up of 17 years. The evaluated predictors included age, sex, diabetes duration, diabetes type, body mass index, insulin use, hypertension, smoking, living region and screen-detected diabetes. In a subcohort of the patients with available information, fasting blood glucose and the history of dyslipidemia were also investigated. In consideration that different causes of death might be associated with differential risk factors, cancer and non-cancer death were analyzed separately.

## Methods

### Study Subjects

The study was approved and supported by an ethics review board of the Department of Health of Taiwan (DOH89-TD-1035). Detailed methods for creating a large nationally representative cohort of patients with diabetes for prospective follow-up of their vital status have been previously described [2,10–12,21,26]. In brief, a total of 256036 patients with diabetes were identified from 66 hospitals and clinics located throughout Taiwan from 1995 to 1998. To create a cohort of 90000 patients for long-term follow-up, 128572 cases from the 256036 patients were randomly selected for questionnaire interview, assuming a predicted response rate of 70%.

A total of 93484 (response rate, 72.7%) patients completed the interview. After excluding 938 patients with missing information for the variables included in analyses, the data of a total of 92546 patients were used for the present study. The information abstracted from the questionnaire for this study included age, sex (men versus women), diabetes type [type 1 versus type 2, type 1 diabetes was defined based on either one of the following two criteria: (1) diabetic ketoacidosis at the onset of diabetes mellitus; and (2) the patients required insulin injection within one year of diagnosis of diabetes], screen-detected diabetes (diabetes diagnosed accidentally through epidemiological screening programs or during visits to medical settings without a history of diabetes, yes versus no), diabetes duration (as a continuous variable), self-reported body weight in kilograms and body height in centimeters, insulin use (yes versus no), hypertension (yes versus no), smoking (current smokers and past smokers versus never smokers) and living region. Body mass index was calculated as body weight in kilograms divided by squared body height in meters. Living regions were defined as urban for the Metropolitan Taipei area (including the city of Taipei and the county of Taipei) and other administratively designated cities across Taiwan or as rural for administratively designated counties and offshore islands.

In a subcohort of the patients, who reported to have visited a physician in recent one month, we also asked patients about the results of fasting blood glucose and the history of dyslipidemia [26]. Only patients who reported continuous levels of fasting blood glucose and the presence or absence of a history of dyslipidemia were included in this subcohort analysis.

### Ascertainment of vital status

As described in detail previously [21], in Taiwan, every resident has a unique identification number, and events like birth, death, marriage, or migration should be registered in the household registration offices. If a person dies, a death certificate should be issued by a physician, and this certificate should be reported to the household registration offices within 30 days as required by law. Data from death certificates, including the unique identification number, date of birth, sex, and date and cause of death, have been computerized since 1971 and can be used for academic research. Causes of death have been coded according to the ninth revision of the International Classification of Diseases (ICD-9) in Taiwan since 1981. After 2009, ICD-10 has been used for the coding of the causes of death in Taiwan. Therefore, ICD-9 was used for classifying the causes of death from 1995 to 2008, and ICD-10 was used after 2009.

Specific causes of death were classified into cancer death (ICD-9: 140–208, ICD-10: C00-C97) and non-cancer death (all other ICD-9 codes excluding 140–208 and all other ICD-10 codes excluding C00-C97). Non-cancer deaths were further classified into diabetes mellitus (ICD-9: 250, ICD-10: E10-E14); heart disease (ICD-9: 390–398,410–414, 420–429, ICD-10: I01-I02.0, I05-I09, I20-I25, I27, I30-I52); stroke (ICD-9: 430–438, ICD-10: I60-I69); hypertension and atherosclerosis (ICD-9: 401–405, 440, ICD-10: I10-I15, I70); nephropathy (ICD-9: 580–589, ICD-10: N00-N07, N17-N19, N25-N27); respiratory disease (ICD-9: 460–519, ICD-10: J00-J99); infection (ICD-9: 001–139, ICD-10: A00-B99); digestive diseases (ICD-9: 520–579, ICD-10: K00-K93); accidents (ICD-9: 800–949, ICD-10: V01-X59, Y85-Y86); suicide (ICD-9: 950–959, ICD-10: X60-X84, Y87.0); and other causes (codes other than the above).

### Statistical analyses

All data were de-identified during statistical analyses. Analyses were conducted using SAS statistical software, version 9.3 (SAS Institute, Cary, NC). *P*-values less than 0.05 were considered statistically significant.

All patients were followed up from recruitment until the end of 2011. Attained age was calculated as the sum of age at entry and the available length of follow-up. Baseline characteristics and attained age for patients who died and patients who survived were compared by Chi-square test for categorical variables and by Student’s t test for continuous variables.

Survival curves for 3 different strata of attained age (<45, 45–64 and ≥65 years) and sex (men and women) were first plotted by the Kaplan-Meier method. For patients who survived a certain stratum of age and entered the next stratum with non-zero survival time, they were treated as right-censored at the previous stratum and as late entry in the next stratum. The logrank test was used to test the significance of the survival difference among the different strata of attained age and sex.

Sex-specific mortality rates were then computed using a person-years denominator for patients with all ages at baseline and for different strata of attained age (<45, 45–49, 50–54, 55–59, 60–64, 65–69, 70–74 and ≥75 years). For patients with all ages at baseline, the person-years of follow-up for each patient were calculated as the duration from the date of recruitment until the end of 2011 for those who were alive or to the date of death for those who died. For the different strata of attained age, if a patient survived a certain stratum of age and entered the next stratum, he/she was treated as right-censored at the previous stratum and as late entry in the following stratum. Mortality rate ratios comparing men versus women were calculated for all ages at baseline and for the different strata of attained age. The International Diabetes Federation estimated that 46.6% of the diabetes patients died under the age of 60 [1]. To clarify whether this could be applied to our patients, the proportion distribution of the age at death in this cohort was calculated for men, women and both sexes, respectively. Additionally, the cause-specific mortality rates by sex and the mortality rate ratios for men versus women were calculated.

Cox proportional hazards models were used to estimate the hazard ratios and their 95% confidence intervals for cancer death and non-cancer death, separately. In these Cox regression models, age was considered as the time-scale and calendar effects were adjusted for by stratifying the model by birth cohort in 5-year intervals as described by Canchola et al. [27]. This method considered that the subjects entered the analysis at their baseline age (left-truncation or late entry) and exited the study at their age of event or censoring [27]. Because of the prolonged period of follow-up of the cohort, the inclusion of the birth cohort in 5-year intervals in modeling accounted for the adjustment for calendar effects. The following independent variables were included in the models: age at baseline, sex, diabetes type, screen-detected diabetes, diabetes duration, body mass index (as a continuous variable), insulin use, hypertension, smoking, and living region (Model I). The models were created for all patients, and sensitivity analyses were conducted after excluding 1197 patients who had been followed up for less than 2 years (Model II) to minimize the potential bias due to illness-induced body weight loss in the calculation of body mass index and in the interpretation of cause-effect relationship. For the subcohort of patients who additionally reported their blood glucose levels and history of dyslipidemia, models were created by including these two additional independent variables (Model III).

To further evaluate whether different categories of body mass index might exert different effects on cancer and non-cancer death, the above-mentioned models I, II and III were also created by using the following cutoffs of body mass index for the definition of underweight, normal, overweight, obesity I and obesity II recommended for Asian populations: <18.5, 18.5–22.9, 23.0–24.9, 25.0–29.9 and ≥30.0 kg/m^2^ [28], using normal weight (i.e., 18.5–22.9 kg/m^2^) as the referent group. Dichotomous analyses were also performed by using the recommended cutoffs for obesity I and obesity II, respectively.

## Results

Table 1 compares the baseline characteristics and the attained age between patients who died and those who survived at the end of follow-up. Approximately 43.5% of the patients died during the 17-year follow-up. Patients who died were characterized by older age at baseline, older attained age, male predominance, longer diabetes duration, lower body mass index, higher percentage with type 1 diabetes, more among those whose diabetes was not screen-detected, and greater prevalence of insulin use, hypertension, smoking, and living in rural areas.

**Table 1:** Characteristics of the cohort

Characteristics	Patients survived		Patients died		*P* value
	*n* or mean	% or SD	*n* or mean	% or SD	
*n*	52317	56.53	40229	43.47	
Age at baseline (years)	56.49	11.85	65.31	10.31	<0.0001
Attained age (years)	71.01	11.83	73.11	10.38	<0.0001
Diabetes duration (years)	6.11	5.83	8.75	7.21	<0.0001
Body mass index (kg/m^2^)	24.88	3.57	24.11	3.65	<0.0001
Sex					
Women	29242	58.63	20634	41.37	<0.0001
Men	23075	54.08	19595	45.92	
Diabetes type					
Type 1	1847	52.92	1643	47.08	<0.0001
Type 2	50470	56.67	38586	43.33	
Insulin use					
No	48176	57.89	35051	42.11	<0.0001
Yes	4141	44.44	5178	55.56	
Hypertension					
No	27324	62.40	16467	37.60	<0.0001
Yes	24993	51.26	23762	48.74	
Smoking					
Never smoker	37926	59.04	26307	40.96	<0.0001
Current smoker	9713	53.08	8586	46.92	
Past smoker	4678	46.71	5336	53.29	
Screen-detected diabetes					
No	35857	53.48	31186	46.52	<0.0001
Yes	16460	64.54	9043	35.46	
Living region					
Rural	26230	54.83	21610	45.17	<0.0001
Urban	25063	58.47	17805	41.53	
Unknown	1024	55.71	814	44.29	

SD: standard deviation

**Fig 1:**
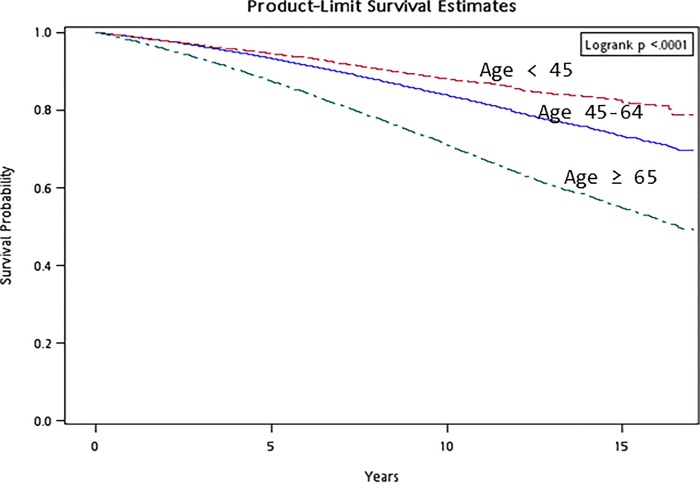
Survival curve for the diabetes cohort by different strata of attained age

**Fig 2:**
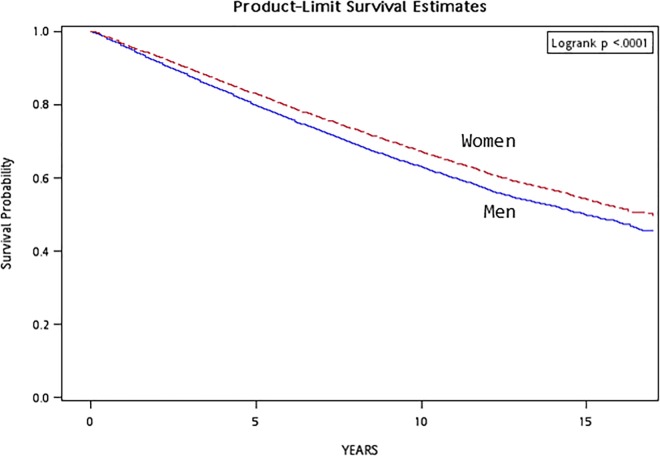
Survival curve for the diabetes cohort by sex

Figs 1 and 2 show the survival curves with regards to different strata of attained age and sex, respectively. Increasing mortality was observed for increasing attained age (Fig 1, logrank test, *P* < 0.0001) and for men than women (Fig 2, logrank test, *P* < 0.0001).

Table 2 shows the sex-specific mortality rates and the mortality rate ratios for men versus women in different strata of attained age and in all ages at baseline, and the proportion distribution of age at death in men, women and both sexes, respectively. In all strata of attained age, men had a significantly higher risk of mortality than their female counterparts. A simple calculation from Table 2 showed that 4385 (2793 men and 1592 women) among the 40229 mortal patients (10.9% for both sexes, 14.3% for men and 7.7% for women) died under the age of 60. Therefore, only 10.9% of the diabetes patients died under the age of 60 in Taiwan, which is much lower than the reported 46.6% by the International Diabetes Federation [1].

**Table 2:** Sex-specific mortality rates and mortality rate ratios for men versus women in different strata of attained age and in all ages at baseline, and the proportion distribution of age at death in men, women and both sexes, respectively

Age	Men				Women				MRR (95% CI)
	*n*	Death	PY	MR	*n*	Death	PY	MR	
Attained age									
<45	4609	319	25673.21	12.43	3277	124	20683.17	6.00	2.07 (1.69–2.54)
45–49	7524	456	25253.04	18.06	5721	180	18374.29	9.80	1.84 (1.56–2.18)
50–54	10745	783	38528.42	20.32	9927	449	33987.63	13.21	1.54 (1.37–1.73)
55–59	14428	1235	49223.08	25.09	15634	839	53821.92	15.59	1.61 (1.47–1.76)
60–64	17251	1899	57699.29	32.91	21770	1710	74898.04	22.83	1.44 (1.35–1.54)
65–69	19355	2768	65497.50	42.26	25959	2821	90838.21	31.06	1.36 (1.29–1.43)
70–74	19524	3685	65469.96	56.29	26127	4057	90933.04	44.62	1.26 (1.21–1.32)
≥75	16987	8450	94297.00	89.61	23044	10454	127855.21	81.76	1.10 (1.06–1.13)
All ages	42670	19595	421641.50	46.47	49876	20634	511391.50	40.35	1.15 (1.13–1.17)
		Men			Women			Both sexes	
		Death	%		Death	%		Death	%
Age at death									
<45		319	1.63		124	0.60		443	1.10
45–49		456	2.33		180	0.87		636	1.58
50–54		783	4.00		449	2.18		1232	3.06
55–59		1235	6.30		839	4.07		2074	5.16
60–64		1899	9.69		1710	8.29		3609	8.97
65–69		2768	14.13		2821	13.67		5589	13.89
70–74		3685	18.81		4057	19.66		7742	19.24
≥75		8450	43.12		10454	50.66		18904	46.99
All ages		19595	100.00		20634	100.00		40229	100.00

*n*: case number followed, PY: person-years, MR: mortality rate (per 1000 PY), MRR: mortality rate ratio, CI: confidence interval

Table 3 shows the cause-specific mortality rates by sex and the mortality rate ratios for men versus women. For the specific causes of death, the mortality rate ratios suggested that men and women had similar mortality rates for nephropathy; women had a higher risk of mortality from diabetes mellitus and hypertension and atherosclerosis; and men had significantly higher risk of mortality from the other specific causes. In general, the mortality rate ratio for all causes of death suggested that men had a 15% higher risk of mortality than women.

**Table 3:** Cause-specific mortality rates by sex and mortality rate ratios for men versus women

Cause of death	ICD-9 (1995–2008)	ICD-10 (2009–2011)	Men (*n* = 42670)			Women (*n* = 49876)			MRR (95% CI)
			*n*	%	MR	*n*	%	MR	
All causes			19595	100.00	46.47	20634	100.00	40.35	1.15 (1.13–1.17)
Cancer	140–208	C00-C97	3900	19.90	9.25	3124	15.14	6.11	1.51 (1.45–1.59)
Non-cancer	ICD-9 excluding 140–208	ICD-10 excluding C00-C97	15695	80.10	37.22	17510	84.86	34.24	1.09 (1.06–1.11)
Diabetes mellitus	250	E10-E14	5319	27.14	12.61	6904	33.46	13.50	0.93 (0.90–0.97)
Heart disease	390–398,410–414, 420–429	I01-I02.0, I05-I09, I20-I25, I27, I30-I52	1851	9.45	4.39	1799	8.72	3.52	1.25 (1.17–1.33)
Stroke	430–438	I60-I69	1633	8.33	3.87	1790	8.68	3.50	1.11 (1.03–1.33)
Hypertension and atherosclerosis	401–405, 440	I10-I15, I70	198	1.01	0.47	290	1.41	0.57	0.83 (0.69–0.99)
Nephropathy	580–589	N00-N07, N17-N19, N25-N27	1183	6.04	2.81	1509	7.31	2.95	0.95 (0.88–1.03)
Respiratory disease	460–519	J00-J99	1573	8.03	3.73	1185	5.74	2.32	1.61 (1.49–1.73)
Infection	001–139	A00-B99	421	2.15	1.00	433	2.10	0.85	1.18 (1.03–1.35)
Digestive disease	520–579	K00-K93	1266	6.46	3.00	1290	6.25	2.52	1.19 (1.10–1.29)
Accidents	800–949	V01-X59, Y85-Y86	544	2.78	1.29	321	1.56	0.63	2.06 (1.80–2.35)
Suicide	950–959	X60-X84, Y87.0	196	1.00	0.46	131	0.63	0.26	1.81 (1.46–2.26)
Other causes	ICD-9 excluding above	ICD-10 excluding above	1511	7.71	3.58	1858	9.00	3.63	0.99 (0.92–1.06)

ICD: International Classification of Diseases, MR: mortality rate (per 1000 PY), MRR: mortality rate ratio, CI: confidence interval

The hazard ratios for cancer death and non-cancer death are shown in Table 4. Insulin use and smoking were significantly predictive for both cancer death and non-cancer death. Diabetes type had a null association with either cancer death or non-cancer death. On the other hand, living in rural areas was significantly associated with a higher risk of non-cancer death but not of cancer death. Screen-detected diabetes, diabetes duration, body mass index, hypertension, fasting glucose and dyslipidemia might show differential impacts on cancer and non-cancer death. Screen-detected diabetes was associated with a lower risk of both cancer and non-cancer death when the analyses were conducted in the original cohort (Models I and II). However, in the subcohort analyses with additional adjustment for fasting glucose and dyslipidemia, screen-detected diabetes seemed to provide a protective effect only for non-cancer death (Model III). Diabetes duration had a null association with cancer death, but was significantly predictive for non-cancer death in all analyses. A higher body mass index was preventive for non-cancer death, but not for cancer death (Models II and III), supporting the emerging concept of “obesity paradox” [29,30], which might be applied to non-cancer death in our patients with diabetes. Hypertension was predictive for non-cancer death in all models, but might be associated with a lower risk of cancer death in the models including all patients in the original cohort (Models I and II).

**Table 4:** Cox regression models for cancer and non-cancer death

Characteristics	Interpretation	Cancer death			Non-cancer death		
		HR	95% CI	*P* value	HR	95% CI	*P* value
Model I. All patients (*n* = 92546)							
Age at baseline	Every 1-year increment	1.040	(1.029–1.050)	<0.0001	1.035	(1.031–1.040)	<0.0001
Sex	Men vs. Women	1.378	(1.295–1.465)	<0.0001	1.034	(1.004–1.064)	0.0242
Diabetes type	Type 1 vs. Type 2	1.010	(0.863–1.182)	0.9020	0.981	(0.923–1.044)	0.5515
Screen-detected diabetes	Yes vs. No	0.796	(0.754–0.840)	<0.0001	0.683	(0.666–0.702)	<0.0001
Diabetes duration	Every 1-year increment	0.999	(0.995–1.003)	0.5624	1.018	(1.017–1.020)	<0.0001
Body mass index	Every 1-kg/m^2^ increment	0.981	(0.974–0.988)	<0.0001	0.955	(0.951–0.958)	<0.0001
Insulin use	Yes vs. No	1.161	(1.052–1.281)	0.0029	1.469	(1.413–1.526)	<0.0001
Hypertension	Yes vs. No	0.905	(0.862–0.950)	<0.0001	1.469	(1.221–1.278)	<0.0001
Smoking	Current vs. Never	1.457	(1.360–1.560)	<0.0001	1.352	(1.307–1.398)	<0.0001
	Past vs. Never	1.223	(1.128–1.326)	<0.0001	1.352	(1.185–1.280)	<0.0001
Living region	Urban vs. Rural	0.954	(0.909–1.001)	0.0545	0.832	(0.814–0.851)	<0.0001
	Unknown vs. Rural	0.882	(0.741–1.049)	0.1555	0.861	(0.797–0.930)	0.0001
Model II. Sensitivity analysis in all patients: Excluding patients followed up <2 years (*n* = 91349)							
Age at baseline	Every 1-year increment	1.000	(0.989–1.010)	0.9314	1.003	(0.998–1.007)	0.2462
Sex	Men vs. Women	1.353	(1.264–1.448)	<0.0001	1.026	(0.994–1.059)	0.1075
Diabetes type	Type 1 vs. Type 2	1.034	(0.865–1.235)	0.7157	0.984	(0.918–1.055)	0.6496
Screen-detected diabetes	Yes vs. No	0.802	(0.756–0.851)	<0.0001	0.685	(0.665–0.705)	<0.0001
Diabetes duration	Every 1-year increment	1.000	(0.996–1.004)	0.9711	1.018	(1.016–1.020)	<0.0001
Body mass index	Every 1-kg/m^2^ increment	0.995	(0.988–1.003)	0.2572	0.964	(0.961–0.968)	<0.0001
Insulin use	Yes vs. No	1.130	(1.012–1.261)	0.0298	1.419	(1.359–1.481)	<0.0001
Hypertension	Yes vs. No	0.906	(0.859–0.956)	0.0003	1.235	(1.204–1.266)	<0.0001
Smoking	Current vs. Never	1.505	(1.396–1.622)	<0.0001	1.378	(1.328–1.429)	<0.0001
	Past vs. Never	1.210	(1.106–1.324)	<0.0001	1.194	(1.143–1.246)	<0.0001
Living region	Urban vs. Rural	0.985	(0.934–1.038)	0.5704	0.844	(0.824–0.865)	<0.0001
	Unknown vs. Rural	0.925	(0.765–1.118)	0.4205	0.921	(0.847–1.000)	0.0510
Model III*. Subcohort patients with fasting glucose and recorded history of dyslipidemia (*n* = 14559)							
Age at baseline	Every 1-year increment	1.024	(0.996–1.053)	0.0936	1.012	(1.000–1.024)	0.0466
Sex	Men vs. Women	1.487	(1.259–1.756)	<0.0001	1.065	(0.984–1.151)	0.1174
Diabetes type	Type 1 vs. Type 2	0.944	(0.642–1.390)	0.7709	1.031	(0.869–1.223)	0.7278
Screen-detected diabetes	Yes vs. No	1.082	(0.942–1.243)	0.2655	0.784	(0.731–0.840)	<0.0001
Diabetes duration	Every 1-year increment	1.082	(0.984–1.004)	0.2492	1.017	(1.013–1.021)	<0.0001
Body mass index	Every 1-kg/m^2^ increment	0.988	(0.969–1.007)	0.2028	0.965	(0.956–0.973)	<0.0001
Insulin use	Yes vs. No	1.344	(1.049–1.722)	0.0193	1.351	(1.219–1.499)	<0.0001
Hypertension	Yes vs. No	0.964	(0.846–1.098)	0.5792	1.259	(1.184–1.340)	<0.0001
Smoking	Current vs. Never	1.581	(1.319–1.895)	<0.0001	1.412	(1.288–1.548)	<0.0001
	Past vs. Never	1.581	(1.027–1.569)	0.0275	1.256	(1.133–1.393)	<0.0001
Living region	Urban vs. Rural	0.887	(0.781–1.009)	0.0681	0.821	(0.773–0.872)	<0.0001
	Unknown vs. Rural	0.554	(0.337–0.913)	0.0203	0.876	(0.726–1.056)	0.1659
Fasting glucose	Every 1-mg/dL increment	1.000	(0.998–1.001)	0.8855	1.003	(1.002–1.003)	<0.0001
Dyslipidemia	Yes vs. No	0.989	(0.831–1.178)	0.9031	1.142	(1.060–1.230)	0.0005

HR: hazard ratio, CI: confidence interval

*Sensitivity analyses in the subcohort after excluding patients who had been followed up for <2 years did not remarkably change the hazard ratios (data not shown).

Smoking: Yes = current smoking + past smoking

Table 5 shows the results of the Cox models using categorical cutoffs of body mass index. It was clearly shown that “obesity paradox” existed only in non-cancer death but had a null effect on cancer death after additional adjustment for fasting glucose and history of dyslipidemia (Model III). The lowest risk of mortality seemed to fall within a body mass index of 25–29.9 kg/m^2^ in the diabetes patients.

**Table 5:** Cox regression models for cancer and non-cancer death comparing various cutoffs of body mass index recommended for Asian populations

Body mass index cutoffs	Interpretation	Cancer death			Non-cancer death		
		HR	95% CI	*P* value	HR	95% CI	*P* value
Model I. All patients (*n* = 92546)							
Asian recommendation	<18.5 vs. 18.5–22.9 kg/m^2^	1.329	(1.162–1.520)	<0.0001	1.579	(1.497–1.665)	<0.0001
	23–24.9 vs. 18.5–22.9 kg/m^2^	0.885	(0.832–0.942)	0.0001	0.788	(0.766–0.811)	<0.0001
	25–29.9 vs. 18.5–22.9 kg/m^2^	0.874	(0.824–0.928)	<0.0001	0.723	(0.704–0.743)	<0.0001
	≥30 vs. 18.5–22.9 kg/m^2^	0.936	(0.843–1.038)	0.2102	0.767	(0.732–0.805)	<0.0001
Obesity I	≥25 vs. <25 kg/m^2^	0.920	(0.876–0.967)	0.0009	0.787	(0.769–0.805)	<0.0001
Obesity II	≥30 vs. <30 kg/m^2^	1.012	(0.918–1.116)	0.8082	0.910	(0.870–0.953)	<0.0001
Model II. Sensitivity analysis in all patients: Excluding patients followed up <2 years (*n* = 91349)							
Asian recommendation	<18.5 vs. 18.5–22.9 kg/m^2^	1.013	(0.852–1.205)	0.8844	1.412	(1.325–1.504)	<0.0001
	23–24.9 vs. 18.5–22.9 kg/m^2^	0.934	(0.872–1.000)	0.0501	0.822	(0.797–0.848)	<0.0001
	25–29.9 vs. 18.5–22.9 kg/m^2^	0.935	(0.877–0.998)	0.0427	0.763	(0.741–0.786)	<0.0001
	≥30 vs. 18.5–22.9 kg/m^2^	1.023	(0.915–1.143)	0.6957	0.802	(0.762–0.845)	<0.0001
Obesity I	≥25 vs. <25 kg/m^2^	0.977	(0.926–1.031)	0.3981	0.823	(0.803–0.844)	<0.0001
Obesity II	≥30 vs. <30 kg/m^2^	1.070	(0.964–1.188)	0.2032	0.929	(0.885–0.976)	0.0034
Model III. Subcohort patients with fasting glucose and recorded history of dyslipidemia (*n* = 14559)							
Asian recommendation	<18.5 vs. 18.5–22.9 kg/m^2^	1.160	(0.771–1.745)	0.4764	1.337	(1.136–1.573)	0.0005
	23–24.9 vs. 18.5–22.9 kg/m^2^	0.869	(0.734–1.028)	0.4764	0.828	(0.767–0.893)	<0.0001
	25–29.9 vs. 18.5–22.9 kg/m^2^	0.956	(0.819–1.115)	0.5651	0.733	(0.682–0.788)	<0.0001
	≥30 vs. 18.5–22.9 kg/m^2^	0.902	(0.684–1.189)	0.4640	0.775	(0.684–0.878)	<0.0001
Obesity I	≥25 vs. <25 kg/m^2^	1.004	(0.884–1.142)	0.9462	0.793	(0.747–0.843)	<0.0001
Obesity II	≥30 vs. <30 kg/m^2^	0.951	(0.734–1.233)	0.7052	0.916	(0.814–1.030)	0.1437

HR: hazard ratio, CI: confidence interval

Models are adjusted for the same covariates as shown in Table 4, but only the results for body mass index are shown here.

## Discussion

The findings support a survival advantage in obese patients with diabetes, especially for non-cancer death (Models I to III, Tables 4 and 5), reconfirming our previous findings after a 12-year follow-up of the cohort [21]. Furthermore, a potential benefit for the screening of diabetes in terms of mortality, especially for non-cancer death, was observed (Table 4). Insulin use is significantly predictive for a higher risk of mortality from either cancer or non-cancer death (Table 4).

In consideration that the non-cancer death grouping in Tables 4 and 5 was very heterogeneous, additional analyses were conducted by restricting the causes of death to those more specific to vascular complications related to diabetes which included those ascribed to diabetes mellitus, heart disease, stroke, hypertension and atherosclerosis and nephropathy as shown in Table 3. It was noted that the hazard ratios for deaths due to these diabetes complications for all the variables were very similar to those estimated for non-cancer death in Tables 4 and 5, respectively. Furthermore, the significance of *P* values remained unchanged for each of the variables (data not shown). These additional analyses suggested that the findings for non-cancer death in Tables 4 and 5 might be more specific to diabetes-related vascular complications. The null association of diabetes duration with cancer death and positive association with non-cancer death (Table 4) also indicated that the non-cancer death might be more specific to diabetes complications which are highly correlated to metabolic control and diabetes duration. On the other hand, even though diabetes patients might suffer from a higher mortality from cancer as reported in an early study of this cohort [11], the link between diabetes and cancer death might not be directly related to the metabolic milieu, but probably through genetic linkage between insulin resistance and cancer.

In a previous study that followed the mortality of the same diabetes cohort for a period of up to 12 years, obesity provided a survival advantage in the Taiwanese patients with diabetes, which was especially significant for non-cancer death [21]. By following the cohort for an additional 5 years in the present study, such an “obesity paradox” was reconfirmed for non-cancer death (Models I to III, Tables 4 and 5) or for the death due to diabetes-related vascular complications as mentioned above (data not shown). The significantly lower risk of cancer death associated with increasing body mass index in Model I of Tables 4 and 5 might indicate a potential bias of illness/cancer-induced body weight reduction (reverse causality), because such “obesity paradox” was no more observed when patients having a short follow-up period of <2 years were excluded in the analysis (Model II, Tables 4 and 5) or when additional confounders such as fasting glucose and history of dyslipidemia were included in the analyses (Model III, Tables 4 and 5). Therefore, “obesity paradox” might only be applicable to non-cancer death or the death due to diabetes-related vascular complications, but not to cancer death. The existence of “obesity paradox” in association with non-cancer death indicates a reconsideration of the necessity and the extent for weight reduction in the obese patients with diabetes.

The mechanisms for a better survival in non-cancer death or diabetes-related vascular complications in diabetes patients with obesity remain to be explored. Hainer and Aldhoon-Hainerová in a recent review article provided several hypothetical explanations for such an “obesity paradox” [31]. First, obese patients who have a higher proportion of abdominal fat are at a higher risk of dying earlier and at a younger age. Therefore, those who have less risky obesity may survive. Second, many elderly patients may have late-onset obesity and therefore, the health risk of obesity may not manifest because of its short duration. Third, metabolically healthy obesity can be seen in the elderly who may have similar body mass index but lower visceral fat or waist circumference than obese insulin-resistant patients. These patients with metabolically healthy obesity did not show an increased risk of all-cause, cardiovascular or cancer mortality in a 15-year follow-up study conducted in Italy [32]. Fourth, body mass index may not be a good indicator for abdominal obesity, especially in the elderly. Fifth, the rate of intra-abdominal fat accumulation decreases with age, and therefore, metabolically inactive storage of peripheral fat may predominate in the elderly and responsible for the “obesity paradox”. Sixth, body mass index may be a better indicator of lean body mass than of body fat. Therefore, a large body mass index in the elderly may represent an increased volume of skeletal muscles rather than an indication of increased total body fatness. On the other hand, a low body mass index may be a surrogate for sarcopenia, which can exacerbate insulin resistance and dysglycemia. Seventh, a lower body mass index may be indicative of the “malnutrition-inflammation complex syndrome”, which is associated with a poor prognosis in patients with chronic heart failure and hemodialysis. Eighth, autopsy studies suggested that severely obese subjects might have been partially protected from the development of diabetic vascular complications via enhanced mobilization of endothelial progenitor cells or decreased production of thromboxane. Ninth, increased caloric intake and weight gain may improve the sensitivity to ghrelin in the hypothalamus and myocardium and thus protect against heart failure and cardiac cachexia. Tenth, obese patients with heart failure produce lower concentrations of tumor necrosis factor-α, which can further be lowered by the production of soluble receptors that bind this factor by subcutaneous adipose tissue.

Whether screening for diabetes may provide a survival advantage for the screen-detected patients is controversial. In a follow-up of the Ely cohort of 4936 people aged 40–65 years and without known diabetes at baseline in the United Kingdom, individuals participated in a diabetes screening program by invitation in 1990–1992 might have a non-significant 21% lower risk of all-cause mortality than the non-invited group while followed up until 1999. However, no significant difference in mortality could be shown between invited and non-invited participants for a later screening conducted in 2000–2003 and followed to 2008 [22]. Further analyses of 92 screen-detected and 60 unscreen-detected patients with diabetes in the Ely cohort suggested that the earlier diagnosis of diabetes by screening did not appear to have an impact on health outcomes [23]. Although the effect of lead time bias can not always be excluded in patients who received a screening program, screen-detected diabetes patients might have represented those who had their diabetes diagnosed at an early stage and early treatment for diabetes might have exerted a beneficial effect on the development and progression of diabetes complications and related comorbidities. Additionally, screen-detected patients might have cared more for their own health and therefore their behavior and attitude towards diseases might be different from unscreen-detected patients who might have cared less for their health and had their diabetes detected at a later stage of the disease. The above reasons could probably explain why screen-detected diabetes was associated with a lower risk of both cancer death and non-cancer death in the study (Models I and II, Table 4).

The definition of diabetes in Taiwan always follows the criteria of the World Health Organization (WHO). Because the cohort was recruited during 1995–1998 and most of the patients had their diabetes diagnosed before the revision of the diagnostic criteria published by the WHO in 1999 [33], the definition of diabetes in this cohort mainly referred to the WHO criteria defined in 1980 with updated revision in 1985 [34]. Accordingly, the definition of diabetes in asymptomatic patients primarily referred to the following criteria of blood glucose levels: 1) fasting plasma glucose levels ≥ 140 mg/dL; and 2) 2-hour after 75-g oral glucose load ≥ 200 mg/dL. In patients with classical symptoms of increased thirst and urine volume, unexplained weight loss, drowsiness and coma, diabetes was always diagnosed by a random plasma glucose ≥ 200 mg/dL [34].

Approximately 10.9% of the patients died under the age of 60 (Table 2). Although this figure is much lower than the estimated 46.6% by the International Diabetes Federation [1], the premature death associated with diabetes strongly implicates an urgent need for aggressive intervention to curb the current trends of diabetes-related death. Early detection of diabetes by screening may be an important method and the present study provided evidence supporting the conduction of such a screening program for diabetes. Because screening for diabetes does not cause any long-term harm, either physically or psychologically [24,25], the benefits of a population-based screening program may probably outweigh the potential harms. Future studies are required for investigating the cost-effectiveness of diabetes screening.

Exogenous insulin use is a potential risk factor for atherosclerosis and cancer [13,18]. However, findings in epidemiological studies have been inconsistent [13,18]. The present study is probably the first to demonstrate a significantly higher risk of mortality from either cancer or non-cancer death associated with insulin use (Models I to III, Table 4). It is worth to point out that insulin use was also associated with a higher risk of hypertension incidence in patients with diabetes [10]. Because hypertension was the most important risk factor for cardiovascular disease in the Taiwanese patients with diabetes [8], it is reasonable to infer that insulin use may increase the development of cardiovascular disease through the effect of hypertension. In some other studies, insulin use also increased the risk of some types of cancer, including non-Hodgkin’s lymphoma [19], breast cancer [35,36] and colorectal and liver cancer [20]. Future investigation is required to clarify whether insulin use may only be associated with some types of cancer but not with others.

Patients living in rural areas have a significantly higher risk of non-cancer death in all analyses (Models I to III, Table 4). Living in rural areas may be viewed as a surrogate for socioeconomic status, or as an indicator for different lifestyle or different accessibility to health care. In the present study, hypertension is consistently predictive for non-cancer death (Models I to III, Table 4), but not for cancer death in the model considering additional adjustment for fasting glucose and history of dyslipidemia (Model III of Table 4). Smoking has been well recognized as an important risk factor for the mortality from a variety of cancer and cardiovascular disease [37]. This is similarly observed for either cancer or non-cancer death (Models I to III, Table 4). In consistent with several previous studies conducted in the USA [38], Italy [39] and Taiwan [11], diabetic men in the present study also show a higher risk of mortality than their female counterparts in different analyses (Tables 1–4, Fig 2). Similar observations of a significantly higher risk of mortality associated with hypertension, smoking and for the male patients indicate the validity of the present study.

Only a small proportion of the patients had type 1 diabetes (*n* = 3490 or 3.8%) in the cohort. In secondary analyses, the exclusion of patients with type 1 diabetes did not change the findings or the conclusions of the study (data not shown). Therefore, the findings of the present study should better be applied to patients with type 2 diabetes. Furthermore, because Asians have a higher proportion of body fat than non-Asians at the same level of body mass index [40], whether the findings of the present study can be generalized to non-Asians remain to be answered.

This study has several strengths. It is population-based, evaluating a large nationally representative sample of patients. The cohort was followed prospectively over a long duration, and the prospective nature allowed us to avoid some of the limitations associated with case-controlled designs, such as selection bias, recall bias, and reverse causality. The ascertainment of patients’ vital statuses by matching with the National Death Certificate Database was complete.

There are also some limitations to this study. First, body mass index was derived from self-reported body weight and height. Although this correlates well with measured data and has been used in epidemiological studies, self-reported body height is always over-reported and weight under-reported, especially at higher body mass index [9]. Second, information on blood glucose and history of dyslipidemia could only be collected in a subcohort of the patients. Third, except for insulin use, this study did not collect information on the use of other anti-diabetic medications, or medications commonly used in the diabetic patients for the treatment of dyslipidemia and hypertension. Fourth, information on changes in body weight, glycemic control and other time-varying variables over time was not available, and it is not possible to evaluate the effects of the changes in these variables on mortality. Fifth, information on cancer stage, which is a major prognostic indicator of mortality, was not available for the study.

## Conclusions

In a prospective follow-up of a nationally representative cohort of patients with diabetes, screen-detected diabetes is associated with a lower risk of mortality, especially for non-cancer death. Insulin use is significantly predictive for mortality from both cancer and non-cancer death. Furthermore, obesity may provide a survival advantage for non-cancer death, and probably not for cancer death.
